# Complete Genome Sequence of West Nile Virus Strains Used for the Formulation of CBER/FDA RNA Reference Reagents and Lot Release Panels for Nucleic Acid Testing

**DOI:** 10.1128/genomeA.00811-14

**Published:** 2014-10-30

**Authors:** Andriyan Grinev, Germán Añez, Maria Rios

**Affiliations:** Laboratory of Emerging Pathogens, Division of Emerging and Transfusion Transmitted Diseases, Office of Blood Research and Review, Center for Biologics Evaluation and Research, U.S. Food and Drug Administration, Bethesda, Maryland, USA

## Abstract

We report the complete sequences of two West Nile virus strains (FDA-Hu02 and NY99) used for the formulation of CBER/FDA RNA reference reagents and lot release panels for use with nucleic acid technology testing.

## GENOME ANNOUNCEMENT

West Nile virus (WNV) (genus *Flavivirus*, family *Flaviviridae*) is the most widely extended arbovirus in the world. WNV transmission involves a bird-mosquito enzootic cycle that can infect humans, horses, and other vertebrates who act mainly as dead-end hosts. WNV appeared for the first time in the Americas in the United States in 1999 and since then it has disseminated extensively within the country and also to other countries on the continent (1). There are two main WNV lineages (lineages I and II), and to date only lineage I is present in the Americas, whereas both lineages have been involved in recent epidemics in Europe (2, 3). During the course of the successive WNV epidemics in the United States, a number of genotypes have emerged since the introduction of the initial NY99 genotype. Currently, all WNV isolates that circulate in the country belong to any of three genotypes: WN02, SW/WN03, and MW/WN06 (2, 4, 5).

Approximately 80% of WNV human infections are asymptomatic. Most of the symptomatic infections present clinically as an influenza-like illness, whereas a small percentage of cases develop neuroinvasive disease, which is a life-threatening condition. There are no vaccines or specific antiviral treatments for WNV (1). WNV is transmissible by blood transfusion and organ transplant (6, 7) and to date there are two FDA-approved nucleic acid testing (NAT) blood screening assays for WNV (8). In 2003, a panel of CBER/FDA reference reagents was prepared using two WNV lineage I strains collected in the United States during the initial years of the WNV epidemic. This material was used to assist in the development of NAT assays for blood screening; it is currently used to verify regulatory compliance in the evaluation of licensed WNV assays prior to its release into the market (lot release).

We describe here the complete sequences of the WNV strains FDA-Hu02 (collected from an asymptomatic infected blood donor in 2002) and NY99 (obtained from an infected flamingo in 1999). Cell culture supernatants from the second passage of the NY99 strain and third passage of the FDA-Hu02 strain in Vero cells were used for total RNA extraction using the QIAamp Viral RNA minikit (Qiagen). Reverse transcription reactions, PCR amplification, sequencing of overlapping PCR products, and sequence analysis were performed as described previously (4).

The total lengths of the genomes of the WNV NY99 and FDA-Hu02 strains are 11,029 and 11,030 nucleotides (nt), respectively. The isolate FDA-Hu02 showed 20 nucleotide mutations plus one insertion at position 10,497 compared to prototype NY99; 5 mutations resulted in amino acid substitutions. We found no differences between the nucleotide sequences of the original NY99 isolate (AF196835) and the NY99 isolate cultivated and sequenced in our laboratory. Phylogenetic analysis classified FDA-Hu02 as belonging to genotype WN02, whereas strain NY99 is the prototype of the NY99 genotype (2).

### Nucleotide sequence accession numbers.

The complete sequence of the WNV strains FDA-Hu02 and NY99 have been submitted to the GenBank under the accession numbers AY646354 and KM083619, respectively.
